# Complications associated with single-level bone transport for the treatment of tibial bone defects caused by fracture-related infection

**DOI:** 10.1186/s12891-023-06527-2

**Published:** 2023-06-23

**Authors:** Kai Liu, Qiyu Jia, Xin Wang, Yemenlehan Bahesutihan, Chuang Ma, Peng Ren, Yanshi Liu, Aihemaitijiang Yusufu

**Affiliations:** 1 Department of Trauma and Microreconstructive Surgery, The First Affiliated Hospital of Xinjiang Medical University, Urumqi, 830054 Xinjiang China; 2 Department of Orthopaedics, The Affiliated Hospital of Southwest Medical University, Luzhou, 650032 Sichuan China

**Keywords:** Bone defects, Bone transport, Complications, External fixator, Ilizarov method, osteomyelitis

## Abstract

**Background:**

The purpose of this study was to report the outcomes of single-level bone transport with a unilateral external fixator for treatment of proximal, intermediate and distal tibial bone defects caused by fracture-related infection (FRI) and compare their complications.

**Methods:**

The clinical records and consecutive X-ray photographs of patients with tibial bone defects treated by single-level bone transport using a unilateral external fixator (Orthofix Limb Reconstruction System) were analyzed retrospectively, from January 2012 to December 2018. Patients were divided into the proximal group (P, n = 19), intermediate group (I, n = 25), and distal group (D, n = 18) according to the location of the tibial bone defect. The Association for the Study and Application of the Method of Ilizarov (ASAMI) standard was applied to assess the bone and functional outcomes and postoperative complications evaluated by the Paley classification.

**Results:**

A total of 62 participants were included in this study, with a median age of 36 ± 7.14 years. **S**ixty patients with tibial bone defects caused by FRI were successfully treated by single-level bone transport using a unilateral external fixator, with a mean bone union time (BUT) of 7.3 ± 1.71 months. According to the ASAMI criteria, there were statistical differences in bone and function results between the three groups (P vs. I vs. D, *P* < 0.001). The excellent and good rate of bone result in the intermediate group was higher than the other (P vs. I vs. D, 73.6% vs. 84% vs. 66.7%), and the excellent and good rate of function result in the proximal group was the highest (P vs. I vs. D, 84.2% vs. 80% vs. 73.3%). Complications were observed in 29 out of 62 patients (46.7%), with pin tract infection being the most common (14.8%), followed by axial deviation (14.8%), muscle contractures (12.7%), joint stiffness (12.7%), and soft tissue incarceration (12.7%). Other complications included delayed consolidation (12.7%), delayed union (6.3%), nonunion (4.2%), and neurological injury (8.5%). Two patients (3.2%) required below-knee amputation due to uncontrollable infection and previous surgery failure.

**Conclusions:**

Pin tract infection was the most common complication in tibial bone transport using an external fixator. Complications of distal tibial bone transport are more severe and occur at a higher rate than in other parts. Axial deviation mostly occurred in the intermediate tibial bone transport.

## Background

With the increasing familiarity of orthopaedic surgeons with distraction osteogenesis principles, the use of bone transport via a unilateral external fixator for the treatment of tibial bone defects caused by fracture-related infection (FRI) is gaining further improvement [1–4]. The satisfactory clinical efficacy of bone transport using a unilateral external fixator has been reported by published studies [3, 5, 6]. While previous studies have certified the clinical efficacy of single-level bone transport, the total treatment period of this technique is long and cumbersome, requiring meticulous patient instruction, and support compliance. Previous studies have noted major complications, including delayed union and docking site nonunion, mostly occurring in bone transport for the treatment of distal tibial bone defect, despite pin tract infection remaining the most common complication with the utilisation of external fixator [1, 7, 8]. The clinical complications and pathological mechanisms of single-level bone transport in treating proximal, intermediate, and distal tibial bone defects caused by FRI have been rarely reported.

The purpose of this study was to report the outcomes of single-level bone transport with a unilateral external fixator for the treatment of proximal, intermediate and distal tibial bone defects caused by FRI and compare their complications.

## Methods

Permission from the Ethics Committee of the First Affiliated Hospital of Xinjiang Medical University was obtained and informed consent was received from all patients. The clinical records and consecutive X-ray photographs of patients with tibial bone defects treated by single-level bone transport using a unilateral external fixator (Orthofix Limb Reconstruction System, Verona, Italy) were analyzed retrospectively, from January 2012 to December 2018.

### Inclusion and exclusion criteria

Inclusion criteria included [9]: patients with a tibial bone defect caused by FRI [10], sinus tract or abscess of affected limbs, adequate bone stalk for Schanz screw insertion, and treated by single-level bone transport using a unilateral external fixator. Adequate bone stalk for Schanz screw insertion was defined as the presence of at least 4 to 6 cm bone segments proximal and distal to the tibia for screw insertion, which were proximal and distal fixed bone segments. Patients with age < 18 years, tibial bone defects caused by tumor resection or congenital limb discrepancy, incomplete medical records, poor compliance, and follow-up time < 20 months were excluded [9].

The demographic data, initial injury, previous treatment, antimicrobial utilization, and culture results of secretions were documented. Physical examinations included evaluation of knee and ankle range of motion (ROM) and soft tissue condition. Inflammatory markers such as C-reactive protein (CRP), white blood cell (WBC) count, procalcitonin, and erythrocyte sedimentation rate (ESR) were measured. Cierny and Mader’s (CM) classification was used to assess the severity of bone infection. All patients were given appropriate intravenous antibiotics for 2 weeks based on the identified bacteria’s sensitivity.

### Surgical technique

The detailed preoperative plan was conducted by experienced surgeons according to X-ray graphs, CT scans, and three-dimensional reconstruction images. Patients were positioned supine on the radiolucent table, under spinal anaesthesia. the affected limb’s necrotic bone and soft tissue were removed until the residual bone showed evidence of punctate cortical hemorrhage (paprika sign), via our previous study [9, 11]. The biopsies and cultures of the lesion were collected during the surgical procedure. The surgical area was flushed with 0.9% saline under low pressure. The gloves of all participating surgeons and surgical instruments were replaced. Antibiotic-impregnated cement spacer (5 g vancomycin per 40 g gentamicin-loaded bone cement, Heraeus, Hanau, Germany) equal in length to the bone defect was then filled into the defect to receive a high level of local antibiotic concentrations. The external fixator was placed in an anteromedial position parallel to the axial force line (i.e., placed on the anteromedial aspect of the calf). Three 4.5-mm-diameter Schanz screws were inserted at the proximal and distal part of the tibia, and two 4.5-mm-diameter Schanz screws were inserted at the transport bone segment [9]. After the external frame and sliding clamps were assembled, the minimally invasive osteotomy was performed using a Gigli saw. The osteotomy was performed in the metaphysis to obtain a sufficient blood supply. The soft tissue defects were repaired using direct suturing with appropriate tension, a locally propelled skin flap, or a vascularized free flap.

### Postoperative management

After 7 to 10 days following DO surgery, the distraction phase was initiated at a rate of 0.5 mm/12 hours. Patients were advised against weight-bearing activities on the second postoperative day but encouraged to engage in full weight-bearing walking during both the distraction and consolidation phases.

Bone regeneration at the distraction area was monitored by radiography every other week during the distraction phase and monthly during the consolidation phase. Patients were given instructions on pin tract care to avoid infection. Removal of the external frame was possible when two-thirds of the circular cortical bone had formed in the distracted area. Patients were advised to refrain from weight-bearing walking for two weeks after external fixator removal and instead use a brace or crutches. Afterwards, full weight-bearing walking was encouraged.

### Data Collection and Outcome evaluation

Data collection was performed by two independent investigators who were residents of the orthopaedics department of our institution but were not involved in the treatment of patients. The postoperative data were documented, including defect size(DS), bone union time (BUT), external fixation time (EFT), and external fixation index (EFI). The tibia was artificially divided into three equal segments: the proximal 1/3, intermediate 1/3, and distal 1/3. Patients were divided into the proximal group (P, n = 19), intermediate group (I, n = 25), and distal group (D, n = 18) according to the location of the tibial bone defect. The appearance of sinus drainage and/or sequestrum was considered to be FRI and classified using the Cierny and Mader (CM) classification. EFI (month/cm) was defined as the ratio of the external fixation time to the length of the bone defect.

Patients were provided with instructions on pin tract care to prevent infection or pin tract loosening, which included washing the pin tract using a swab with 0.9% saline solution. As part of the rehabilitation process, patients were encouraged to begin performing active and passive range of motion exercises for the affected limb without bearing weight. Partial weight-bearing walking was allowed on the second day after surgery, and the use of a walker or crutches was permitted starting from the second postoperative week. After discharge, patients in this cohort were followed at 1, 3, 6, 9, 12, 18, and 24 months. The ASAMI criteria were applied to assess the bone and functional outcomes, and postoperative complications were evaluated by the Paley classification [12]. In the management of complications, minors such as mild pin tract infection and pain were successfully resolved through dressing changes and oral medication, without the need for additional surgery. Major complications were resolved with additional surgery, such as axial deviation that was corrected by adjusting the sliding clamps of the external fixator under local anaesthesia. Sequelae were categorized as remaining unresolved complications, such as deformity, skin paresthesia, and limited joint movement. The main factors affecting bone transport procedures were compared among the three groups, including demographic data, type of initial fracture, previous treatment, duration of FRI, DS, BUT, EFT, EFI, bone and functional results, and complications.

### Statistical analysis

Data were captured in a Microsoft Excel spreadsheet (Redmond, Washington, USA) and analysis was performed by the SPSS 20.0 software package (Chicago, Illinois, USA). The variance analysis was used for the comparison of quantitative variables between the three groups. Comparisons of categorical variables between three groups were performed using the Kruskal-Wallis test. Comparisons between the two groups were performed using the chi-square test or t-test. Statistical significance was *P* < 0.05.

## Results

This study included 62 participants with a mean age of 36 ± 7.14 years, consisting of 47 males (75.8%) and 15 females (24.1%). The mean postoperative follow-up time was 27.8 ± 6.37 months. The mechanisms of initial tibia fracture were road traffic accidents (46.7%), falling from height (14.5%), direct trauma (22.5%), or other (16.1%). Based on Cierny and Mader’s (CM) classification, there were 19 cases of type II, 32 cases of type III, and 11 cases of type IV. The mean duration of FRI in the cohort was 30.9 ± 3.86 months. There were no statistically significant differences in age, gender, type of fracture, initial injury, and duration of FRI among the three groups (*P* > 0.05, Table 1).

**Table 1:** Comparison of demographic data in three groups

Variables	Proximal (n = 19)	Intermediate (n = 25)	Distal (n = 18)	P value
Age (years)	36(25–50)	35(26–45)	36(28–45)	0.862
Gender (male, female)	15M4F	19M6F	13M5F	0.894
Type of fracture (open, closed)	16O3C	18O7C	15O3C	0.540
Treatment of initial injury (CREF, ORIF, both of two)	9C4O6B	15C7O3B	12C3O3B	0.369
Duration of FRI (month)	30(23–37)	31(22–40)	31(24–41)	0.300

CREF, closed reduction and external fixation; FRI, fracture-related infection; ORIF, open reduction and internal fixation

Microbiological analysis showed positive results in 53 cases (85.4%), with phenotypically indistinguishable pathogens identified by culture from at least two separate deep tissue/implant specimens. Staphylococcus aureus was found in 36 patients (67.9%), Staphylococcus epidermidis in 9 patients (16.9%), and Escherichia coli in 8 patients (13.2%). Statistical differences were observed in WBC (16.24 ± 4.51 10^9^/L vs. 7.39 ± 1.86 10^9^/L), CRP (19.38 ± 7.35 mg/L vs. 5.62 ± 1.24 mg/L), ESR (28.14 ± 6.37 mm/h vs. 11.92 ± 4.66 mm/h), and procalcitonin (0.67 ± 0.09 ng/mL vs. 0.23 ± 0.06 ng/mL) between the preoperative and first postoperative month (*P* < 0.05).

**Fig. 1:**
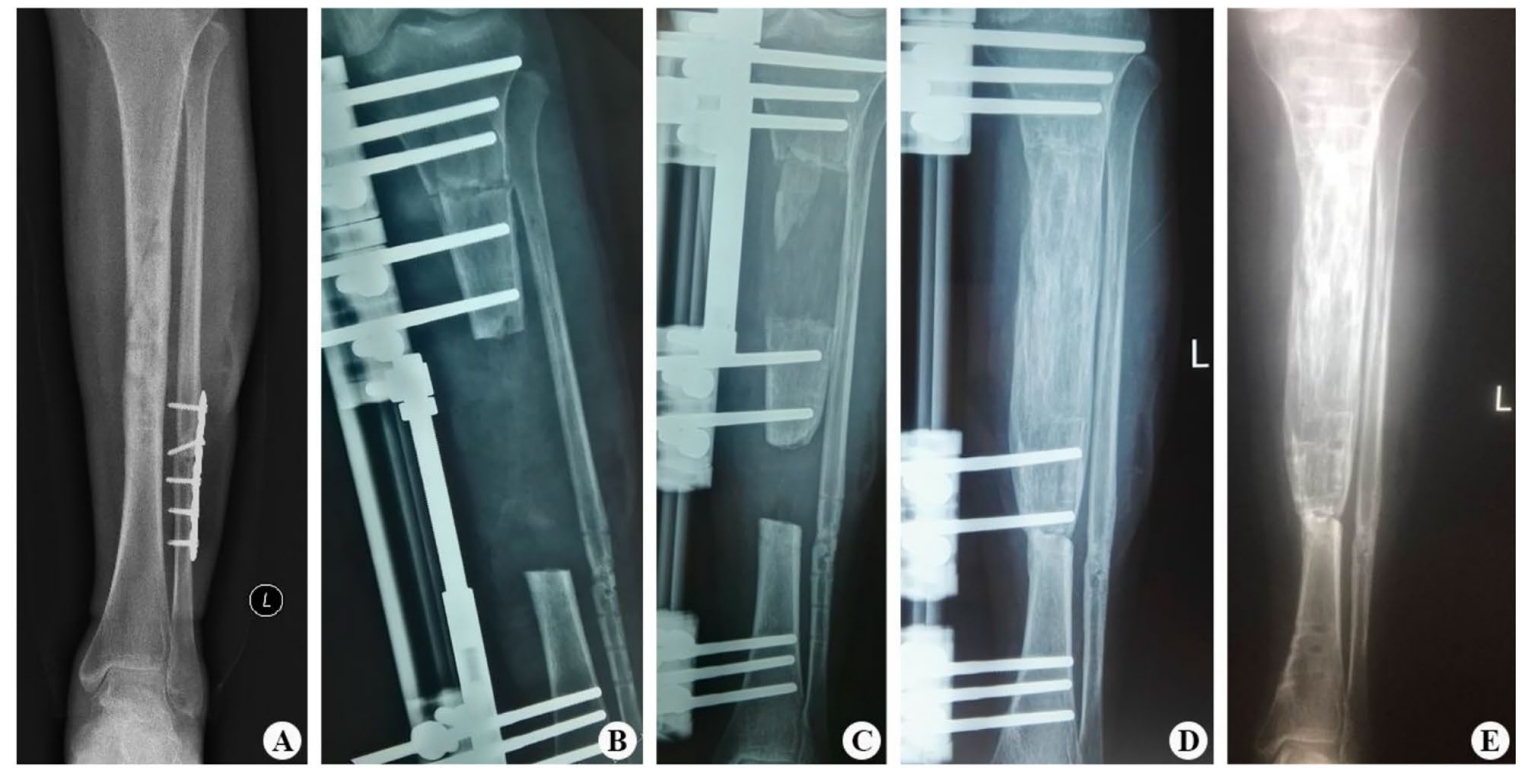
A 47-year-old female with post-traumatic osteomyelitis of the left tibia was treated with single-level bone transport using Orthofix external fixator. (**A**) X-ray of left tibia before single-level bone transport surgery. (**B**) Postoperative X-ray of the left tibia showed that DS after debridement was almost 7.4 cm. (**C**) X-ray at 4 weeks in the distraction phase. (**D**) Bone transport was completed with good consolidation, and docking union was achieved in the 9th postoperative month. (**E**) The external frame was removed with an excellent bone result after 11 postoperative months

**Fig. 2:**
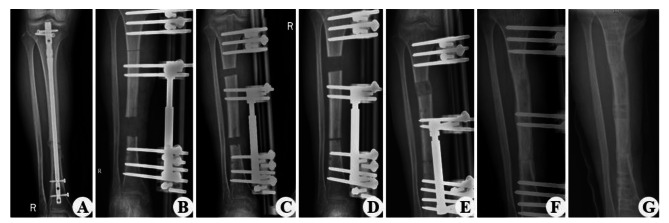
A 37-year-old female with nonunion of the right tibia was treated by single-level bone transport. (**A**) Nonunion of the right tibia after fracture reduction surgery. (**B**) DS after debridement was almost 4.9 cm. (**C, D**) Distraction phase. (**D**) Bone transport was completed with a satisfactory bone union in the 7th postoperative month. (**E, F**) Consolidation phase. (**G**) The external frame was removed after 8 postoperative months

**Fig. 3:**
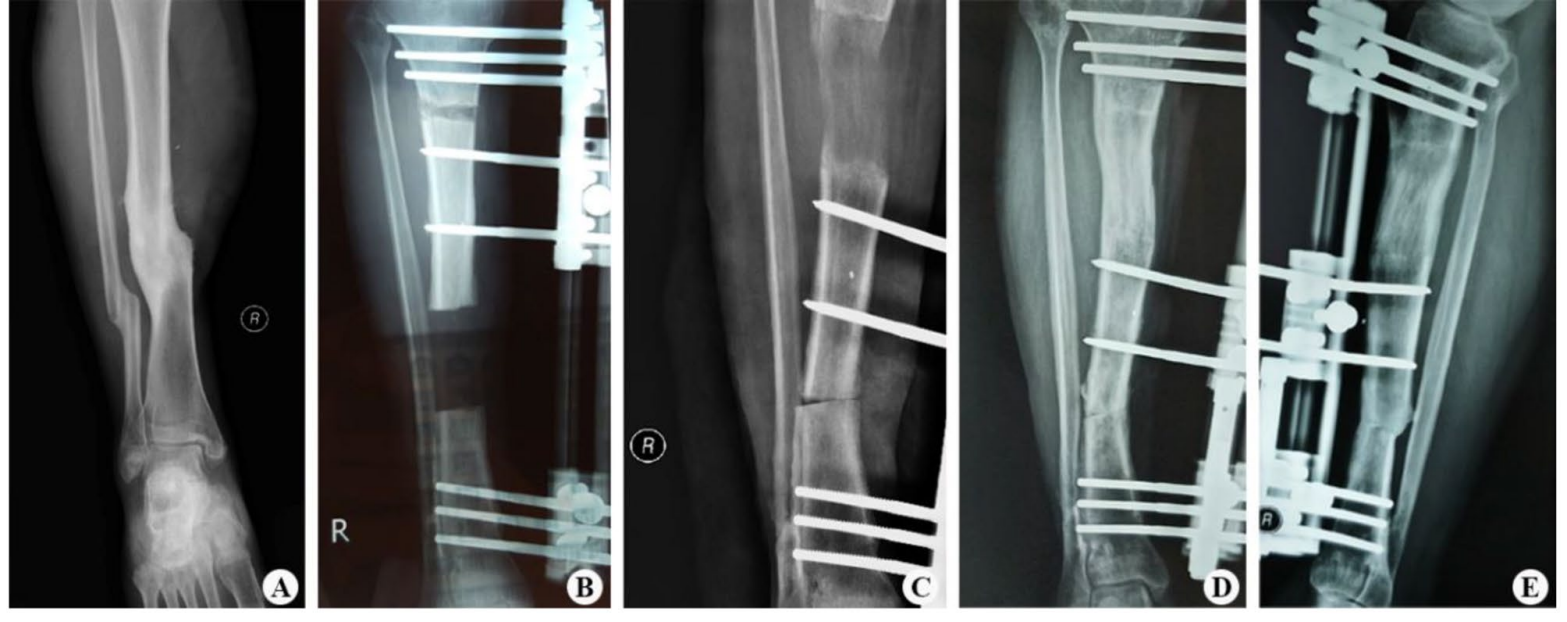
A 39-year-old male with nonunion of the right tibia caused by FRI was treated with single-level bone transport. (**A**) Nonunion of the right tibia caused by FRI. (**B**) X-ray showed that DS after debridement was almost 5.3 cm at the intermediate part of the tibia. (**C**) Third postoperative weeks at the distraction phase. (**D, E**) Bone transport was completed with a satisfactory bone union after 6 postoperative months

Sixty patients with tibial bone defects caused by FRI were successfully treated by single-level bone transport using a unilateral external fixator, with a mean bone union time of 7.3 ± 1.71 months. The mean length of the bone defect was 4.6 ± 1.83 cm, and the bone union was achieved in 60 cases (96.7%). Soft tissue loss was treated with direct sutures of appropriate tension in 19 patients (30.6%), local propulsive skin flaps in 32 patients (51.6%), and vascularized free flaps in 11 patients (17.7%), with no necrosis observed. There were no statistically significant differences in DS, BUT, EFT, EFI, and follow-up time (P > 0.05, see Table 2) among the three groups. Details of the entire procedure described in this study were presented in Figs. 1, 2 and 3.

**Table 2:** Comparison of postoperative data in three groups

Variables	Proximal (n = 19)	Intermediate (n = 25)	Distal (n = 18)	P value
DS (cm)	4.8(3.0-6.6)	4.8(3.6–7.4)	4.9(3.9–5.9)	0.574
BUT(month)	7.0(6–8)	7.0(6–8)	7.1(6–8)	0.072
EFT (month)	8.0(7–9)	7.9(7–9)	7.8(7–8)	0.559
EFI (month/cm)	1.68(1.29–2.18)	1.71(1.20–2.34)	1.70(1.15–2.21)	0.963
Follow-up time(month)	25(20–33)	27(21–42)	24(22–31)	0.362

BUT, bone union time; DS, defect size; EFT, external fixation time; EFI, external fixation index

According to the ASAMI criteria, there were significant differences in bone and functional results among the three groups (P vs. I vs. D, *P* < 0.001). The intermediate group had a higher rate of excellent and good bone results compared to the other groups (P vs. I vs. D, 73.6% vs. 84% vs. 66.7%), while the proximal group showed the highest rate of excellent and good function results (P vs. I vs. D, 84.2% vs. 80% vs. 73.3%), as summarized in Table 3. Additionally, the distribution of complications across the three groups was documented in Table 4. According to the Paley classification, statistical significance was observed for minor complications per patient, major complications per patient, and sequelae per patient (*P* < 0.05) between the intermediate and distal groups (Table 5).

**Table 3:** Comparison of bone and function outcomes in three groups according to ASAMI criteria

ASAMI	Location	Excellent	Good	Fair	Poor	Failure
Bone grade^#^	Proximal	6	8	2	3	-
	Intermediate	4	17	3	1	
	Distal	3	9	3	1	2
Function grade^*^	Proximal	3	13	2	1	-
	Intermediate	9	11	3	1	-
	Distal	4	7	3	2	2

P, proximal; I, intermediate; Distal, D

# P vs. I p = 0.834, I vs. D p < 0.001, P vs. D p < 0.001

* P vs. I p = 0.768, I vs. D p = 0.032, P vs. D p < 0.001

**Table 4:** The postoperative complications of three groups

Complication	Proximal (n = 19)	Intermediate (n = 25)	Distal (n = 18)
Pin tract infection or pin loosening	3	2	2
Muscle contractures	1	3	2
Joint stiffness	2	0	4
Axial deviation	1	4	2
Soft tissue incarceration	1	3	2
Neurological injury	1	0	1
Delayed consolidation	1	2	3
Delayed union	0	1	2
Nonunion	0	0	2
Re-fracture	0	0	0

**Table 5:** Comparison of incidence of complications in three groups according to Paley classification

Indicators	Proximal(n = 19)	Intermediate (n = 25)	Distal (n = 18)	P value		
				P vs. I	I vs. D	P vs. D
Docking Site Revision	1	1	4	-	-	-
Minor complications (per patient)	0.61	0.68	0.79	0.596	0.002	< 0.001
Major complications (per patient)	0.21	0.36	0.61	0.934	< 0.001	0.011
Sequelae (per patient)	0.15	0.04	0.16	0.012	< 0.001	0.632

P, proximal; I, intermediate; Distal, D

Complications were observed in 29 of 62 patients (46.7%). The rate of complication in the distal group (0.77 per patient) was higher than in the proximal (0.26 per patient) and intermediate (0.4 per patient). Pin tract infections in 12 patients were managed with dressing changes, while 7 axial deviations were corrected by radiological adjustments of the external fixator under local anaesthesia. Muscle contractures in 4 patients were treated with tension-release surgery, and delayed consolidation or delayed union was addressed through bone grafting at the docking site. Soft tissue incarceration in 6 patients was treated with resection surgery to clear the barriers. Two cases (3.2%) experienced failure due to the recurrence of uncontrollable infection (CM classification IV type C), necessitating several additional surgeries and resulting in severe limitation of ankle motion. For these two patients, amputation was performed to preserve the knee joint and allow for convenient prosthesis use.

## Discussion

The treatment of infected tibial defects is not only an orthopaedic problem but required a multidisciplinary diagnostic and therapeutic approach. Bone transport, based on the principles of distraction osteogenesis, is a practical technique that can be used to treat various complex skeletal diseases. This technique offers advantages such as artificially-controlled velocity and angle of the external frame, complete elimination of infection, satisfactory bone union, and earlier postoperative rehabilitation [2, 13–18]. In this study, 62 tibial bone defects caused by FRI were treated with single-level bone transport, resulting in a total union rate of 96.7%, which confirmed the practical efficacy of this technique.

Pin tract infection is still the most common complication when the external fixator is applied, with an incidence range of 6-96.6% [19, 20]. A recent study by Testa et al. [21] described the successful treatment of tibial bone defects caused by FRI in 26 patients using the Ilizarov technique, with a complication rate of 20%, primarily due to pin tract infection and/or wire and/or screw breakage. The incidence of pin tract infection in this study was 11.2%, mostly occurring in the proximal group. To minimize the risk of pin tract infection, it is essential to use a pin sleeve during insertion to avoid the entanglement of subcutaneous tissue and nerves. Additionally, screws should pass through both cortices perpendicularly to the medullary canal to ensure their stability and prevent loosening and infection.

In bone transport of the intermediate group, tissue-associated complications are more common due to abundant soft tissues that can invade the distraction area. This may be attributed to the anatomy of this part with abundant soft tissues, which may invade the distraction area causing complications. Physiologically, the rich tissue and blood vessels surrounding the bone provide nutrients such as oxygen and calcium, facilitating new bone formation in the distraction area. However, this favourable environment also poses a higher risk of soft tissue incarceration. A recent study [22] has proposed that bone transport combined with the masquelet technique can effectively manage lower limb bone defects, which may reduce the occurrence of soft-tissue-related complications. In our study, 11 patients received free flaps to cover soft tissue loss without soft tissue-related complications. We believe that preoperative-designed flaps may not result in unnecessary tissue. Biweekly X-rays and Doppler sonography of the affected limb can also assist in monitoring bone regeneration in the distraction area. When there is difficulty in sliding the transport bone segment or sclerotic bone appears at the docking site, it suggests that there may be soft tissue incarceration. Besides, minimally invasive osteotomy with periosteum preservation and induced membrane followed by bone transport can both effectively reduce the occurrence of soft tissue-related complications. These techniques prevent soft tissue invasion into the distraction area by protecting or inducing the periosteum. Furthermore, increasing the length of the external rail can produce stable force torque, and maintaining a 4–5 cm distance between sliding clamps can prevent axial deviation [23, 24]. However, further certification is needed to confirm the efficacy of combined methods.

In the distal group, a higher incidence of major complications such as joint stiffness, delayed union, or nonunion was observed. This could be due to poor vascularity in this area, which is also part of the transition zone of the tibial anatomical structure. Fortunately, bone union was achieved in the majority of cases (81.8%) after additional surgery involving bone graft at the docking site for delayed consolidation/union. Two patients (3.2%) underwent below-knee amputation due to uncontrollable infection (CM classification IV type C), failed previous surgery (debridement, correction, etc.), and severe ankle limitation. Similar results were documented in the study of Yin et al. [25], the amputations were 4%, similar to 2.9% reported by Papakostidis et al. [26] and 5.2% reported by Iliopoulous et al. [27] for bone defects caused by FRI. When infection cannot be controlled with antibiotic therapy and multiple previous surgeries, amputation surgery may be considered.

Via recent research, Aktuglu et al. [13] reported a complication rate of 1.22 per patient. In this study, the rate of complication was 0.77 per patient in the distal group, which was the highest among the three groups. Our previous study found that patients with major complications often had an initial injury history of open fractures, which significantly increased the risk of FRI occurrence [28]. In this cohort, the mean duration of FRI was 30.9 ± 3.86 months, leaving a large bone defect after debridement and increasing the difficulty of repairing procedures. During the consolidation phase, we observed another interesting phenomenon between complications. Delayed union or nonunion occurred mostly in patients with axial deviation or soft tissue incarceration, while joint stiffness occurred mainly in patients with proximal or distal tibial bone defects. We suggest that axial deviation and soft tissue incarceration may create difficulty in distracting the transport bone segment, leading to eccentric angles and sclerotic bone at its docking site, which can cause nonunion. Furthermore, fixation across joints is often required to treat bone defects adjacent to joints using bone transport, which may necessitate early initiation of joint range of motion exercises to reduce the incidence of joint stiffness.

Several potential limitations may affect the results of this study. First, this study was conducted retrospectively with a single-center design. Second, there is no unified algorithm for the management of complications in different locations of tibial bone transport, which may obscure the correlation between complications. Third, there is a lack of comparison with the postoperative complications of other limbs treated with bone transport. Thus, a prospective multi-center study with large samples is crucial for the clinical application of bone transport.

## Conclusion

Pin tract infection was the most common complication in tibial bone transport using an external fixator. Complications of distal tibial bone transport are more severe and occur at a higher rate than in other parts. Axial deviation mostly occurred in the intermediate tibial bone transport. A better understanding of the incidence and etiology of complications was important for this technique to be optimally applied.

## Data Availability

The data sets generated and analyzed during the current study are not publicly available due to restrictions on ethical approvals involving patient data and anonymity but can be obtained from the corresponding author as reasonably required.
